# Defining the optimal target-to-background ratio to identify positive lymph nodes in prostate cancer patients undergoing robot-assisted [^99m^Tc]Tc-PSMA radioguided surgery: updated results and ad interim analyses of a prospective phase II study

**DOI:** 10.1007/s00259-024-06789-5

**Published:** 2024-06-11

**Authors:** Leonardo Quarta, Elio Mazzone, Donato Cannoletta, Armando Stabile, Simone Scuderi, Francesco Barletta, Vito Cucchiara, Luigi Nocera, Antony Pellegrino, Daniele Robesti, Riccardo Leni, Paolo Zaurito, Giorgio Brembilla, Francesco De Cobelli, Ana Maria Samanes Gajate, Maria Picchio, Arturo Chiti, Francesco Montorsi, Alberto Briganti, Giorgio Gandaglia

**Affiliations:** 1 Division of Oncology/Unit of Urology, Gianfranco Soldera Prostate Cancer Lab, Urological Research Institute (URI), IRCCS San Raffaele Scientific Institute, Milan, Italy; 2 Department of Radiology, IRCCS San Raffaele Scientific Institute, Milan, Italy; 3 Department of Nuclear Medicine, IRCCS San Raffaele Scientific Institute, Milan, Italy; 4 Vita-Salute San Raffaele University, Via Olgettina 58, 20132 Milan, Italy

**Keywords:** Prostate cancer, Prostate-specific membrane antigen, Radioguided surgery, Lymph node dissection, Staging, Lymph node metastases

## Abstract

**Introduction:**

Prostate-specific membrane antigen radioguided surgery (PSMA-RGS) might identify lymph node invasion (LNI) in prostate cancer (PCa) patients undergoing extended pelvic lymph node dissection (ePLND). The optimal target-to-background (TtB) ratio to define RGS positivity is still unknown.

**Materials & methods:**

Ad interim analyses which focused on 30 patients with available pathological information were conducted. All patients underwent preoperative PSMA positron emission tomography (PET). 99m-Technetium-PSMA imaging and surgery ([^99m^Tc]Tc-PSMA-I&S) was administered the day before surgery. In vivo measurements were conducted using an intraoperative gamma probe. Performance characteristics and implications associated with different TtB ratios were assessed.

**Results:**

Overall, 9 (30%) patients had LNI, with 22 (13%) and 80 (11%) positive regions and lymph nodes, respectively. PSMA-RGS showed uptakes in 12 (40%) vs. 7 (23%) vs. 6 (20%) patients for a TtB ratio ≥ 2 vs. ≥ 3 vs. ≥ 4. At a per-region level, sensitivity, specificity and accuracy for a TtB ratio ≥ 2 vs. ≥ 3 vs. ≥ 4 were 72%, 88% and 87% vs. 54%, 98% and 92% vs. 36%, 99% and 91%. Performing ePLND only in patients with suspicious spots at PSMA PET (n = 7) would have spared 77% ePLNDs at the cost of missing 13% (n = 3) pN1 patients. A TtB ratio ≥ 2 at RGS identified 8 (24%) suspicious areas not detected by PSMA PET, of these 5 (63%) harbored LNI, with one pN1 patient (11%) that would have been missed by PSMA PET. Adoption of a TtB ratio ≥ 2 vs. ≥ 3 vs. ≥ 4, would have allowed to spare 18 (60%) vs. 23 (77%) vs. 24 (80%) ePLNDs missing 2 (11%) vs. 3 (13%) vs. 4 (17%) pN1 patients.

**Conclusions:**

PSMA-RGS using a TtB ratio ≥ 2 to identify suspicious nodes, could allow to spare > 50% ePLNDs and would identify additional pN1 patients compared to PSMA PET and higher TtB ratios.

## Introduction

Prostate-specific membrane antigen (PSMA) is a surface protein that is highly overexpressed on most prostate cancer (PCa) cells. Recent studies support the role of PSMA ligand imaging in improving preoperative staging. Beside preoperative PSMA positron emission tomography (PET) for staging purposes, a role for intraoperative surgical guidance using this tracer has been proposed [1, 2]. PSMA radioguided surgery (PSMA-RGS) is a procedure which implies the use of dedicated gamma probes to detect PSMA ligands labeled with gamma emitting radionuclides such as 99m-technetium-PSMA imaging and surgery ([^99m^Tc]Tc-PSMA-I&S) with the aim to provide immediate feedback to the surgeon on the presence of tumoral cells [1–6]. Initial data confirmed the safety and feasibility of this approach in patients with primary and recurrent PCa [7, 8]. This technique improves the ability to remove metastatic lymph nodes and to detect nodal micro-metastases (i.e., ≤ 5 mm in size) that might be missed by pre-surgical imaging [9, 10]. The intrinsic spatial resolution of RGS might be higher than that of PET imaging, thus allowing for the intraoperative detection of nodal micro-metastases. However, the performance characteristics of PSMA-RGS strongly depend on the definition of suspicious nodes adopted. A target-to-background (TtB) ratio ≥ 2 has been proposed [5]. However, this threshold was based on studies that focused on men treated in the recurrence setting [5, 7, 11]. Determining the optimal TtB ratio to define pathological uptake during RGS, which might prompt the decision to remove suspicious tissue or perform an extended pelvic lymph node dissection (ePLND), needs to be assessed. Our objective was to systematically assess the performance characteristics and clinical implications of different TtB ratios (≥ 2 vs. ≥ 3 vs. ≥ 4) and to compare them with preoperative PSMA PET, [^99m^Tc]Tc-PSMA-I&S single-photon emission computed tomography/computed tomography (SPECT/CT), and histopathological findings at final pathology. This would assess the optimal definition of positivity for PSMA-RGS, thus providing relevant information for clinicians adopting this technique.

## Materials and methods

### Study design

The study cohort comprised patients diagnosed with localized PCa (cTanyN0M0 at abdominal CT or Magnetic Resonance Imaging [MRI] and bone scan [BS]) treated with robot-assisted radical prostatectomy (RARP) with a risk of lymph node invasion (LNI) ≥ 5% according to the Briganti 2019 nomogram [12] and thus candidate for RARP with ePLND, between June 2021 and July 2023. All patients have been enrolled in a phase II, single-institution, national, non-comparative, non-randomized, prospective study (NCT04832958). The prospective trial was funded through a competitive grant by the Italian Ministry of Health (Giovani Ricercatori GR2018-12368369) and aims to enroll a final cohort of 100 PCa patients. Main exclusion criteria include the receipt of neoadjuvant therapies, prior PCa treatments, or involvement in other experimental trials. All participants signed an informed consent and received treatment at a tertiary referral center. Surgery was performed by three high-volume surgeons who had performed more than 100 cases of RARP at the time of the study initiation. Our ad interim analyses focused on the first 30 patients with available pathologic information.

### Preoperative PSMA PET and [^99m^Tc]Tc-PSMA-I&S SPECT/CT

**Fig. 1:**
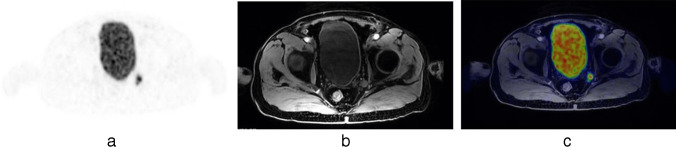
[^68^Ga]Ga-PSMA-11 PET/MRI scan identified a suspicious lymph node at the level of the left obturatory region, as shown in PET (**a**), MRI (**b**) and PET/MRI (**c**) axial view

**Fig. 2:**
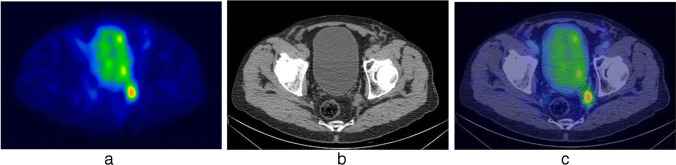
[^99^mTc]Tc-PSMA-I&S SPECT/CT scan identified a suspicious lymph node at the level of the left obturatory region, as shown in SPECT (**a**), CT (**b**) and SPECT/CT (**c**) axial view

Before surgery, patients underwent PSMA PET for preoperative staging. Evaluation of all PSMA PET scans at our center was performed by two experienced high-volume nuclear medicine physicians. Although the original protocol planned for all patients to receive [^68^Ga]Ga-PSMA-11 PET/MRI or PET/CT, the study was amended to allow for sparing this procedure in men who were already staged with [^18^F]F-PSMA-1007 PET/MRI or PET/CT before study enrollment. This adjustment aimed at reducing radio-exposure and was necessary due to the increasing adoption of preoperative PSMA PET for staging purposes. Information obtained at PSMA PET (activity [MBq], prostate and pelvic lymph nodes uptake) performed before study inclusion was collected. The results of the preoperative PSMA PET did not alter the initially planned treatment. Following approval from the Italian Medicines Agency, [^99m^Tc]Tc-PSMA-I&S was prepared using a synthesis kit (piCHEM, Raaba-Grambach, Austria) and administered a day prior to RARP with ePLND (20 h before surgery; median activity: 734 MBq). SPECT/CT imaging occurred 270 min after the administration of [^99m^Tc]Tc-PSMA-I&S to document tracer uptake and served as a quality control. Positive findings on PSMA PET and [^99m^Tc]Tc–PSMA-I&S SPECT/CT were defined as the presence of any uptake in the pelvic and/or retroperitoneal nodes (Figs. 1 and 2). All images were reviewed by two dedicated nuclear medicine physicians.

### Surgical technique

**Fig. 3:**
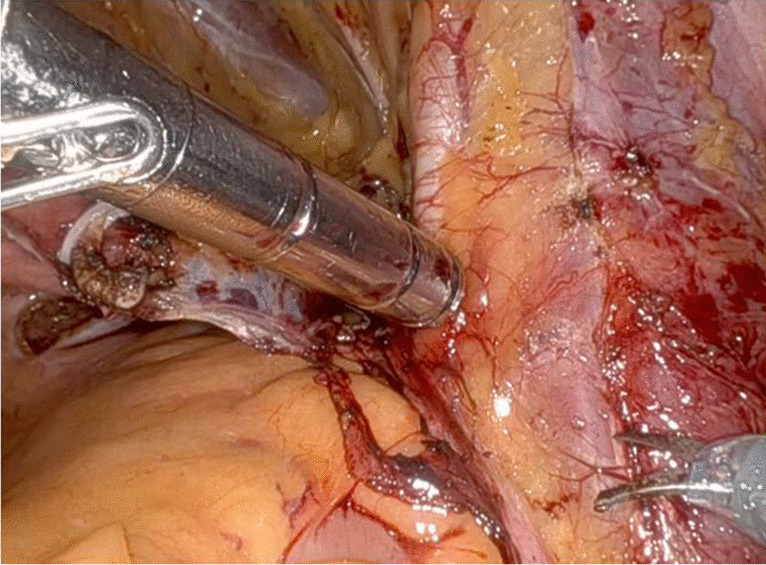
Real-time intraoperative use of the Drop-In gamma probe (Crystal Drop-In Probe; Crystal Photonics, Berlin, Germany) for in vivo measurements at the level of the right external iliac region during PSMA-RGS

All surgeries were conducted via a transperitoneal approach [13] utilizing the Da Vinci Xi® robotic surgical system by three expert surgeons who followed a standardized procedure. A commercially available, CE-marked Drop-In gamma probe (Crystal Drop-In Probe; Crystal Photonics, Berlin, Germany) was inserted through a 15-mm auxiliary port positioned above the right iliac crest. This gamma probe was employed for real-time intraoperative measurements (Fig. 3) to detect potential metastatic sites in the internal iliac, external iliac and obturatory regions. In case of suspicious preoperative imaging or in patients with very-high preoperative LNI risk (Briganti 2019 nomogram > 30%), intraoperative measurements using the Drop-In gamma probe was extended up to the common iliac (above the ureteric crossing), presacral, and retroperitoneal regions [14]. A control unit provides both auditory and numerical feedback in response to [^99m^Tc]Tc-PSMA-I&S activity. We defined a positive finding as any lesion with a TtB ratio at least twice that of the background reference, specifically, the fatty tissue covering the psoas muscle (TtB ratio ≥ 2). Any positive lesion with a TtB ratio exceeding double that of the background reference was surgically removed. After excision, ex vivo gamma measurements were conducted [3]. An anatomically defined ePLND [15] and RARP were then performed [13].

### Statistical analysis

This is an interim analysis after 30 cases aimed at defining the optimal TtB ratio for the identification of nodal metastases. Continuous variables were presented as medians, and categorical variables as proportions. Sensitivity, specificity, positive (PPV) and negative (NPV) predictive values of PSMA-RGS in comparison to the definitive pathology, which represented the gold standard, were computed using contingency tables for both per-patient and per-region analyses. Exact binomial confidence limits were calculated for test sensitivity, specificity, and PPV and NPV, and diagnostic accuracy was defined as the proportion of all tests that give a correct result [16]. To optimize the definition of positivity at PSMA-RGS, different TtB ratios were compared with preoperative imaging exams and with final pathological results. Specifically, TtB ratios ≥ 2, ≥ 3 and ≥ 4 were explored to assess the correspondence with preoperative imaging and with the highest diagnostic accuracy for the identification of positive nodes at final pathology. We investigated the clinical implications of PSMA PET and different TtB ratios, through the number of positive nodes identified and potential ePLNDs spared. Lastly, we compared the maximum diameter of nodal metastases at final pathology among radioactive negative and positive lesions according to the identified optimal TtB ratio to assess the impact of the LNI diameter on PSMA-RGS performance characteristics. All statistical assessments were executed employing R (version 3.6.3).

## Results

### Patients characteristics

The characteristics of patients included in our study are shown in Table 1. Overall, 37% (n = 11) of the patients had EAU intermediate-risk, 53% (n = 16) were classified as high-risk, and 10% (n = 3) had locally advanced PCa. A total of 2 (7%) vs 12 (40%) vs 13 (43%) vs 3 (10%) had respectively ISUP grade group (GG) 2 vs. 3 vs. 4 vs. 5 PCa. The estimated risk for LNI based on the Briganti 2019 nomogram was 18% (IQR: 7–53%). The median number of nodes removed was 22 (IQR:18–31). Nine (30%) patients had LNI at final pathology. Overall, 174 lymph nodal regions including 707 nodes were resected.

**Table 1:** Patients demographics, clinical and pathological characteristics

Characteristic	(n = 30)
Age (years), median (IQR)	68 (62–70)
BMI (kg/m^2^), median (IQR)	25 (23–26)
CCI (Age adjusted), n (%)	
≤ 2	17 (56)
> 2	13 (44)
PSA at biopsy (ng/mL), median (IQR)	8.5 (4.6–16)
PSA density (MRI) (ng/mL/mL), median (IQR)	0.28 (0.18–0.48)
Clinical stage, n (%)	
cT1	9 (30)
cT2	15 (50)
> cT2	6 (20)
Prostate biopsy approach, n (%)	
Systematic	12 (40)
MRI targeted + systematic	18 (60)
Prostate biopsy cores, median (IQR)	
Number of overall cores	15 (14–16)
Number of positive cores	8 (6–10)
Number of systematic cores	12 (12–14)
Number of positive systematic cores	6 (3–8)
Number of MRI targeted cores	3 (2–3)
Number of positive MRI targeted cores	2 (1–3)
Biopsy Grade Group, n (%)	
2 (3 + 4)	2 (7)
3 (4 + 3)	12 (40)
4 (4 + 4)	13 (43)
5 (4 + 5 or 5 + 4)	3 (10)
EAU risk group, n (%)	
Localized, intermediate risk	11 (37)
Localized, high risk	16 (53)
Locally advanced	3 (10)
Risk of LNI according to the Briganti 2019 nomogram (median, IQR)	18 (7–53)
[^68^ Ga]Ga-PSMA-11 PET/MRI and PET/CT, n of positive patients (%)	
Number of patients	22 (100)
Median activity, MBq (IQR)	155 (120–203)
Prostate	22 (100)
Pelvic lymph nodes	2 (9)
[^18^F]F-PSMA-1007 PET/MRI and PET/CT, n of positive patients (%)	
Number of patients	8 (100)
Median activity, MBq (IQR)	294 (258–360)
Prostate	8 (100)
Pelvic lymph nodes	5 (63)
[^99m^Tc]Tc-PSMA-I&S SPECT/CT, n of positive patients (%)	
Median activity, MBq (IQR)	734 (730–738)
Prostate	29 (97)
Pelvic lymph nodes	6 (20)
Tumor stage, n (%)	
pT2c	9 (30)
pT3a	15 (50)
pT3b	6 (20)
Pathological Grade Group, n (%)	
3 (4 + 3)	8 (27)
4 (4 + 4)	12 (40)
5 (4 + 5 or 5 + 4)	10 (33)
Surgical margins, n (%)	
Negative	24 (80)
Positive	6 (20)
Number of removed nodes (median, IQR)	22 (18–31)
Pathological N stage (%)	
pN0	21 (70)
pN1	9 (30)

*IQR*: Interquartile Range; *BMI*: Body Mass Index; *CCI*: Charlson Comorbidity Index; *MRI*: Multiparametric Resonance Imaging; *EAU*: European Association of Urology

### Preoperative PSMA PET and [^99m^Tc]Tc-PSMA-I&S SPECT/CT results

Overall, 7 (23%) patients demonstrated pathologic nodal uptake at PSMA PET, of these, 6 patients (86%) were positive at preoperative [^99m^Tc]Tc-PSMA-I&S SPECT/CT. When compared to final pathology, on a per-patient analysis, PSMA PET showed sensitivity, specificity, PPV and NPV of 67% (95%CI: 30–93%), 95% (95%CI: 76–100%), 86% (95%CI: 42–100%) and 87% (95%CI: 66–97%), respectively. After [^99m^Tc]Tc-PSMA-I&S administration, there were no negative adverse events. [^99m^Tc]Tc-PSMA-I&S SPECT/CT demonstrated lower sensitivity (55%) and same specificity (95%) compared to PSMA PET (Tables 2 and 3).

**Table 2:** Contingency table and diagnostic accuracy on per-patient analysis over a total of 30 patients (a) and on per-region analysis over a total of 174 anatomical lymph nodal regions (b) according to preoperative PSMA PET results

	Pathology positive	Pathology negative
a)		
PSMA PET positive	6	1
PSMA PET negative	3	20
Sensitivity	67% (30–93%)	
Specificity	95% (76–100%)	
Positive Predictive Value	86% (42–100%)	
Negative Predictive Value	87% (66–97%)	
Accuracy	86% (69–96%)	
b)		
PSMA PET positive	11	14
PSMA PET negative	11	138
Sensitivity	50% (28–72%)	
Specificity	91% (85–95%)	
Positive Predictive Value	44% (24–65%)	
Negative Predictive Value	93% (87–96%)	
Accuracy	86% (80–90%)

**Table 3:** Contingency table and diagnostic accuracy on per-patient analysis over a total of 30 patients (a) and on a per-region analysis over a total of 174 anatomical lymph nodal regions (b) according to preoperative 99mTc-PSMA-I&S SPECT/CT results

	Pathology positive	Pathology negative
a)		
99mTc-PSMA-I&S SPECT/CT positive	5	1
99mTc-PSMA-I&S SPECT/CT negative	4	20
Sensitivity	55% (21–86%)	
Specificity	95% (83–100%)	
Positive Predictive Value	83% (48–100%)	
Negative Predictive Value	83% (63–95%)	
Accuracy	83% (68–96%)	
b)		
99mTc-PSMA-I&S SPECT/CT positive	12	3
99mTc-PSMA-I&S SPECT/CT negative	10	149
Sensitivity	54% (28–72%)	
Specificity	98% (93–99%)	
Positive Predictive Value	80% (48–93%)	
Negative Predictive Value	93% (88–96%)	
Accuracy	92% (86–95%)	

### Performance characteristics of different TtB ratios for positive uptake at PSMA-RGS

Overall, 22 anatomical regions (13%) were positive with 80 (11%) nodal metastases at final pathology. At PSMA-RGS, when using a TtB ratio ≥ 2, the drop-in probe detected suspicious nodes in 33 locations (19%) in 12 patients (40%) at in vivo evaluation. Of these, 16 (48%) contained PCa. Overall, a TtB ratio ≥ 2 identified 8 (24%) additional suspicious areas in the pelvic nodal region which were not previously identified by preoperative PSMA PET. Among these additional suspicious areas, 5 (63%) contained positive nodes at pathology. Sensitivity, specificity, PPV and NPV of a TtB ratio ≥ 2 at a per-region analysis were 72% (95%CI: 50–89%), 88% (95%CI: 83–89%), 48% (95%CI: 31–66%), and 96% (95%CI: 91–98%) with a diagnostic accuracy of 87% (95%CI: 81–91%). Sensitivity, specificity, PPV and NPV of TtB ratio ≥ 2 at a per-patient analysis were 78% (95%CI: 40–96%), 76% (95%CI: 53–92%), 58% (95%CI: 28–85%), and 89% (95%CI: 65–99%) with a diagnostic accuracy of 77% (95%CI: 58–90%).

When using a TtB ratio ≥ 3, the drop-in probe detected suspicious nodes in 15 (9%) locations at in vivo evaluation in 7 (23%) patients. Of these, 12 (80%) contained PCa whilst 3 (20%) contained no cancer. Using a TtB ratio ≥ 3, PSMA-RGS did not detect 11 (44%) suspicious areas in the pelvic nodal region which were identified by preoperative PSMA PET. All of these (100%) resulted negative at final pathology. Moreover, a TtB ratio ≥ 3 identified one additional suspicious area which had positive nodes at final pathology, missed by preoperative PSMA PET. Sensitivity, specificity, PPV and NPV of PSMA-RGS at a per-region analysis were 54% (95%CI: 32–76%), 98% (95%CI: 94–100%), 80% (95%CI: 52–96%), and 93% (95%CI: 89–97%) with a diagnostic accuracy of 92% (95%CI: 88–96%). Sensitivity, specificity, PPV and NPV of a TtB ratio ≥ 3 at a per-patient analysis were 66% (95%CI: 30–93%), 95% (95%CI: 76–100%), 86% (95%CI: 42–100%), and 87% (95%CI: 66–97%) with a diagnostic accuracy of 87% (95%CI: 69–96%).

When using a TtB ratio ≥ 4, the drop-in probe detected suspicious nodes in 9 (5%) locations at in vivo evaluation in 6 (20%) patients. Of these, 8 (89%) contained PCa whilst only 1 (11%) contained no cancer. Using a TtB ratio ≥ 4, PSMA-RGS did not identify 16 (64%) suspicious area in the pelvic nodal region which was detected by preoperative PSMA PET. Of these, 3 (19%) had positive nodes at final pathology. Sensitivity, specificity, PPV and NPV of PSMA-RGS at a per-region analysis were 36% (95%CI: 17–59%), 99% (95%CI: 96–100%), 89% (95%CI: 52–100%), and 91% (95%CI: 86–95%), with a diagnostic accuracy of 91% (95%CI: 86–95%). Sensitivity, specificity, PPV and NPV of a TtB ratio ≥ 4 at a per-patient analysis were 55% (95%CI: 21–86%), 95% (95%CI: 76–100%), 83% (95%CI: 36–100%), and 83% (95%CI: 63–95%) with a diagnostic accuracy of 83% (95%CI: 65–94%). Detailed detection rates are reported in Tables 4 and 5.

**Table 4:** Contingency table and diagnostic accuracy on per-region analysis over a total of 174 anatomical lymph nodal regions dissected in 30 patients according to different definitions of PSMA-RGS positivity at in vivo only evaluation

	Pathology positive	Pathology negative
Positivity ≥ 2 target-to-background ratio		
PSMA-RGS positive	16	17
PSMA-RGS negative	6	135
Sensitivity	72% (50–89%)	
Specificity	88% (83–89%)	
Positive Predictive Value	48% (31–66%)	
Negative Predictive Value	96% (91–98%)	
Accuracy	87% (81–91%)	
Positivity ≥ 3 target-to-background ratio		
PSMA-RGS positive	12	3
PSMA-RGS negative	10	149
Sensitivity	54% (32–76%)	
Specificity	98% (94–100%)	
Positive Predictive Value	80% (52–96%)	
Negative Predictive Value	93% (89–97%)	
Accuracy	92% (88–96%)	
Positivity ≥ 4 target-to-background ratio		
PSMA-RGS positive	8	1
PSMA-RGS negative	14	151
Sensitivity	36% (17–59%)	
Specificity	99% (96–100%)	
Positive Predictive Value	89% (52–100%)	
Negative Predictive Value	91% (86–95%)	
Accuracy	91% (86–95%)

**Table 5:** Contingency table and diagnostic accuracy on per-patient analysis over a total of 30 patients according to different definitions of PSMA-RGS positivity at in vivo evaluation

	Pathology positive	Pathology negative
Positivity ≥ 2 target-to-background ratio		
PSMA-RGS positive	7	5
PSMA-RGS negative	2	16
Sensitivity	78% (40–96%)	
Specificity	76% (53–92%)	
Positive Predictive Value	58% (28–85%)	
Negative Predictive Value	89% (65–99%)	
Accuracy	77% (58–90%)	
Positivity ≥ 3 target-to-background ratio		
PSMA-RGS positive	6	1
PSMA-RGS negative	3	20
Sensitivity	66% (30–93%)	
Specificity	95% (76–100%)	
Positive Predictive Value	86% (42–100%)	
Negative Predictive Value	87% (66–97%)	
Accuracy	87% (69–96%)	
Positivity ≥ 4 target-to-background ratio		
PSMA-RGS positive	5	1
PSMA-RGS negative	4	20
Sensitivity	55% (21–86%)	
Specificity	95% (76–100%)	
Positive Predictive Value	83% (36–100%)	
Negative Predictive Value	83% (63–95%)	
Accuracy	83% (65–94%)	

### Clinical implications associated with the adoption of PSMA PET

The use of PSMA PET imaging to select ePLND candidates in case of positive spots would have spared 77% ePLNDs (n = 23) at the cost of missing 13% (n = 3) patients with LNI. On a per-region analysis, this corresponded to 149 (86%) nodal regions spared at the cost of missing 11 (7%) positive nodal regions (Table 2).

### Clinical implications associated with the adoption of different TtB ratios

The use of a TtB ratio ≥ 2 allowed to spare 60% ePLNDs (n = 18) at the cost of missing 11% (n = 2) patients with LNI. On a per-region analysis, this results into 141 (81%) nodal regions spared at the cost of missing only 6 (4%) positive nodal regions. Additionally, a TtB ratio ≥ 2 allowed to identify one patient (11%) with LNI who was missed by preoperative PSMA PET. The use of a TtB ratio ≥ 3 allowed to spare 77% ePLNDs (23 patients) at the cost of missing 13% (n = 3) patients with LNI. On a per-region analysis, this corresponded to 159 (91%) nodal regions spared at the cost of missing 10 (6%) positive nodal regions. The use of a TtB ratio ≥ 4 allowed to spare 80% ePLNDs (24 patients) at the cost of missing 17% (n = 4) patients with LNI. On a per-region analysis, this corresponded to 165 (95%) nodal regions spared at the cost of missing 14 (8%) positive nodal regions (Tables 4 and 5). Notably, TtB ratios ≥ 3 and ≥ 4 did not allow to identify additional patients with LNI missed by preoperative PSMA PET.

### Correlation between TtB ratio ≥ 2 and maximum diameter of nodal metastases

**Fig. 4:**
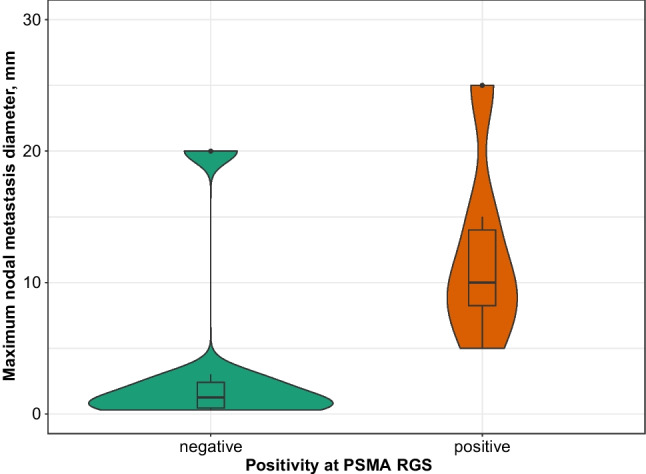
Distribution of maximum diameter of nodal metastasis stratified according to PSMA-RGS positivity based on TtB ratio ≥ 2

In patients with confirmed pN1 disease at final pathology, median maximum diameter of the nodal metastases was 4 mm (IQR 1–10 mm). Notably, when comparing pathological evaluation of pN1 patients with radioactive negative vs. positive lesions according to a TtB ratio ≥ 2, the maximum diameter of metastatic lesions was significantly smaller in false negative findings (10 vs. 1.2 mm, p=0.01), with no metastases smaller than 3 mm identified at PSMA-RGS (Fig. 4).

## Discussion

The current study represents the first attempt to define the optimal TtB ratio for identifying nodal metastases during RARP with ePLND in PCa patients undergoing PSMA-RGS. Previous studies evaluated patients undergoing PSMA-RGS for recurrent PCa [5, 7] and proposed a TtB ratio ≥ 2 for intraoperative positivity during PSMA-guidance. However, this has never been validated in the context of primary PCa. The presence of significantly higher background correlated with the presence of the primary tumor and the entire prostate during surgery may limit the generalizability of this definition. Given recent studies supporting the safety and effectiveness of PSMA-RGS to identify positive nodes during ePLND performed at the time of RARP, a systematic assessment of which is the optimal TtB ratio to define suspicious lymph nodes is urgently needed. In the face of such a paucity of data, we performed ad interim analyses of a prospective phase II study and we tested different TtB ratios to identify the most accurate in identifying positive nodes and their clinical implications. A TtB ratio ≥ 3 exhibited the highest accuracy with perfect correspondence with preoperative PSMA PET at a per-patient analysis. Nonetheless, both preoperative PSMA PET and the use of a TtB ratio ≥ 3 were characterized by a substantial risk of missing positive nodes at final pathology. Conversely, a TtB ratio ≥ 2 definition allowed to correctly identify one additional patient with LNI at the cost of performing less than 15% more ePLNDs.

Many authors are nowadays proposing to avoid an ePLND in patients with negative preoperative PSMA PET [17]. Our results suggest that using PSMA-RGS and removing the nodes only in men with a positive uptake at intraoperative measurements would result in a small number of additional ePLNDs increasing the ability to remove positive lymph nodes. Of note, PSMA-RGS was confirmed as a safe procedure without any complications which could be directly related to the injection of the tracer or the use of the drop-in gamma probe. The sensitivity of PSMA-RGS remains suboptimal with up to 11% patients with LNI missed even when using the lowest TtB ratio. Several reasons might explain these findings. First, although adopting a TtB ratio ≥ 2 might allow for detecting more nodal metastases which could express low volume of PSMA, resulting in fewer positive nodes lost at the cost of removing more negative regions, PSMA expression is heterogeneous both in the primary tumor and in nodal metastases [18]. Immunohistochemical (IHC) analyses on lymph nodes specimens might allow to determine which PSMA expression level in nodal metastases could correlate with the ideal TtB ratio. Second, nodal micro-metastases which are often undetected by preoperative PSMA PET, might be missed, in a smaller proportion, also by RGS due to its intrinsic spatial resolution [19]. Our analysis demonstrated a substantial difference in the diameter of lesions detected by PSMA-RGS vs. those that were missed, where this approach was only partially able to overcome the dimensional limitation of PSMA PET, where no nodal metastases smaller than 3 mm have been identified. Finally, the suboptimal sensitivity of PSMA-RGS even when using a TtB ratio ≥ 2 might be related to the surgeon’s learning curve for the use of the probe and to the presence of confounding sources of [^99m^Tc]Tc-PSMA-I&S activity. In fact, the signal emitted by lymph nodes and measured with the probe, might be interfered by prostate and rectal background noise and by the bladder and ureters, due to the urinary excretion of the radiotracer [4]. Given these issues, it urges the necessity to assess the learning curve for the optimal use of the probe during PSMA-RGS. Moreover, new radiotracers with lower renal excretion and tissues uptake around lymph nodes are needed to limit the background noise and maximize the detection rate of this technique.

From a clinical standpoint, our findings suggest that using PSMA-RGS with a TtB ratio ≥ 2 could further reduce the residual number of patients with false negative findings at preoperative PSMA PET. In men with negative PSMA PET, the use of RGS and sparing an ePLND in men with negative findings at both procedures could further reduce the risk of missing [20]. Similarly, when considering patients with a positive preoperative PSMA PET or those with very high-risk disease, using RGS with a TtB ratio ≥ 2 can still have a role to identify additional suspicious areas outside the standard ePLND template which should be excised.

Our study is not devoid of limitations. First, our results originate from a high-volume tertiary referral center with extensive experience in both RARP and radiotracers; the generalizability of our findings to other settings is not warranted. Second, we presented preliminary results based on first cohort of 30 patients who underwent PSMA-RGS. Therefore, data and results will be further corroborated when achieving the completion of our study protocol. Finally, while the initial protocol intended to administer [^68^Ga]Ga-PSMA-11 PET/MRI or PET/CT to all patients at our center, the study was amended to include patients staged with [^18^F]F-PSMA-1007 PET/MRI or PET/CT before enrolling in the study. This might have introduced an element of heterogeneity in the preoperative assessment.

## Conclusions

We systematically addressed the performance characteristics and clinical implications of PSMA PET and different TtB ratios to identify positive nodes during PSMA-RGS. Preoperative PSMA PET and a TtB ratio ≥ 3 resulted in increased accuracy and reduced instances of false positives in comparison to a TtB ratio ≥ 2. However, they were associated with an unacceptable proportion of positive nodes and patients with LNI missed and an only modest increase in the number of ePLNDs spared. We recommend that the TtB ratio ≥ 2 should be considered to limit the risk of false negative findings and to improve the sensitivity of PSMA-RGS.

## Data Availability

The datasets generated during and/or analysed during the current study are available from the corresponding author on reasonable request.
